# *Giardia duodenalis* Infection Reduces Granulocyte Infiltration in an *In Vivo* Model of Bacterial Toxin-Induced Colitis and Attenuates Inflammation in Human Intestinal Tissue

**DOI:** 10.1371/journal.pone.0109087

**Published:** 2014-10-07

**Authors:** James A. Cotton, Jean-Paul Motta, L. Patrick Schenck, Simon A. Hirota, Paul L. Beck, Andre G. Buret

**Affiliations:** 1 Department of Biological Sciences, University of Calgary, Calgary, Alberta, Canada; 2 Inflammation Research Network, University of Calgary, Calgary, Alberta, Canada; 3 Host-Parasite Interactions, University of Calgary, Calgary, Alberta, Canada; 4 Department of Biochemistry and Molecular Biology, University of Calgary, Calgary, Alberta, Canada; 5 Department of Medicine, University of Calgary, Calgary, Alberta, Canada; 6 Department of Physiology and Pharmacology, University of Calgary, Calgary, Alberta, Canada; 7 Department of Immunology, Microbiology and Infectious Diseases, University of Calgary, Calgary, Alberta, Canada; University of Calgary, Canada

## Abstract

*Giardia duodenalis (syn. G. intestinalis, G. lamblia)* is a predominant cause of waterborne diarrheal disease that may lead to post-infectious functional gastrointestinal disorders. Although *Giardia*-infected individuals could carry as much as 10^6^ trophozoites per centimetre of gut, their intestinal mucosa is devoid of overt signs of inflammation. Recent studies have shown that in endemic countries where bacterial infectious diseases are common, *Giardia* infections can protect against the development of diarrheal disease and fever. Conversely, separate observations have indicated *Giardia* infections may enhance the severity of diarrheal disease from a co-infecting pathogen. Polymorphonuclear leukocytes or neutrophils (PMNs) are granulocytic, innate immune cells characteristic of acute intestinal inflammatory responses against bacterial pathogens that contribute to the development of diarrheal disease following recruitment into intestinal tissues. *Giardia* cathepsin B cysteine proteases have been shown to attenuate PMN chemotaxis towards IL-8/CXCL8, suggesting *Giardia* targets PMN accumulation. However, the ability of *Giardia* infections to attenuate PMN accumulation *in vivo* and how in turn this effect may alter the host inflammatory response in the intestine has yet to be demonstrated. Herein, we report that *Giardia* infection attenuates granulocyte tissue infiltration induced by intra-rectal instillation of *Clostridium difficile* toxin A and B in an isolate-dependent manner. This attenuation of granulocyte infiltration into colonic tissues paralled decreased expression of several cytokines associated with the recruitment of PMNs. *Giardia* trophozoite isolates that attenuated granulocyte infiltration *in vivo* also decreased protein expression of cytokines released from inflamed mucosal biopsy tissues collected from patients with active Crohn’s disease, including several cytokines associated with PMN recruitment. These results demonstrate for the first time that certain *Giardia* infections may attenuate PMN accumulation by decreasing the expression of the mediators responsible for their recruitment.

## Introduction

*Giardia duodenalis* (syn. *G. intestinalis, G. lamblia*) is a non-invasive, small intestinal protozoan parasite infecting a variety of animal species, including humans. *Giardia duodenalis* is currently subdivided into eight distinct genetic assemblages, with only assemblages A and B being infective to humans [1], [2]. Some reports suggest that these assemblages may be unique *Giardia* species, but much controversy remains on this topic [3], [4]. This parasite induces a response within its host that may cause malabsorptive diarrheal disease ([5]). Recent evidence also indicates that giardiasis can lead to the development of post-infectious disorders, in the intestine as well as extra-intestinally, via mechanisms that remain obscure ([6], [7]). During the acute stage of the infection, parasite loads can exceed 10^6^ trophozoites/cm of gut but the intestinal mucosa of most *Giardia*-infected patients is devoid of overt signs of inflammation [8]. These observations are counter-intuitive, considering that *Giardia* infections increase small intestinal permeability via several mechanisms and, therefore, may indeed facilitate the translocation of luminal antigens into underlying host tissues [9]–[11]. However, microscopic duodenal inflammation has been observed in some patients with chronic assemblage B *Giardia* infections following metronidazole treatment [12]. *Giardia* assemblage B infections *in vivo* have been shown to induce small intestinal inflammation [13]. Therefore, it remains to be determined whether *Giardia* infections modulate pro-inflammatory responses within the intestinal mucosa and if these events are assemblage or isolate specific.

Transmission of *Giardia* infections occurs via ingestion of infectious cysts in contaminated food or water, or directly via the fecal-oral route (reviewed in [14]). This route of infection is adopted by many gastrointestinal pathogens, and as a result, *Giardia* co-infections may occur. Indeed, *Giardia* infections have been reported to occur concurrently with *Ascaris sp.* [15], *Cryptosporidium sp.* [16], *Helicobacter pylori* [16], [17], *Vibrio cholerae* [18], *Salmonella* sp. [19] and rotavirus [18], [20], [21]. Many of these pathogens are known to promote inflammatory responses and simultaneously induce diarrheal disease within their hosts. A recent report suggested that Tanzanian children infected with *Giardia* have reduced incidence rates of diarrheal disease and fever, while also displaying lower serum C-reactive protein (CRP) levels [22]. *Giardia* infections have also been shown to attenuate symptoms of diarrheal disease during rotavirus infection [20]. The mechanisms remain unclear, it has been suggested that *Giardia* infections may create an advantageous environment for other co-infecting gastrointestinal pathogens [18]. Contradictorily, a separate study suggests that *Giardia* infections may instead enhance symptoms of diarrheal disease during rotavirus infection [16].

Polymorphonuclear leukocytes (PMNs) are heavily involved in inflammatory responses, and essential to host defence against many bacterial and fungal pathogens. Indeed, genetic mutations or drug therapy that result in defective PMN function make individuals highly susceptible to life-threatening infections [23]–[25]. These cells have been implicated in a variety of inflammatory disorders, in the gut and beyond [26], [27]. PMN recruitment into intestinal tissues is a multistep process involving egression from bone marrow and circulation, migration through host tissues, and transepithelial migration [28]–[30]. Moreover, multiple mediators are known to promote tissue accumulation of PMNs. During acute inflammatory responses, granulocyte colony stimulating factor (G-CSF) promotes PMN bone marrow egression and increases the number of circulating PMNs [31], [32]. Production of interleukin-17A (IL-17A) by tissue-resident cells increases circulating G-CSF levels and promotes subsequent PMN bone marrow egression [32]–[34]. PMN chemokines containing a consecutive glutamate-leucine-arginine sequence (ELR+ chemokines) in their N-terminal region, such as CXCL1, CXCL2, and IL-8/CXCL8, are produced by a variety of cells within the intestinal mucosa. ELR+ chemokines are involved in multiple processes of PMN tissue recruitment, including bone marrow egression [31], exit from the blood stream [30], [35], [36], and migration through host tissues [37], [38].

Previous research has suggested that *Giardia* excretory/secretory products induce IL-8/CXCL8 expression in intestinal epithelial cells [39]. However, we and others have demonstrated that *Giardia* trophozoites attenuate IL-8/CXCL8 secretion from *in vitro* epithelial monolayers [40], [41]. Our research further demonstrated that *Giardia* cathepsin B family proteases contributed to the degradation of IL-8/CXCL8 and attenuated PMN chemotaxis [41]. As PMN accumulation contributes to the development of diarrheal disease via several different mechanisms [28], [42]–[44], we hypothesized that during a *Giardia* infection, the attenuation of diarrheal disease or the creation of a favourable environment for other co-infecting gastrointestinal pathogens may stem, at least partially, from *Giardia’*s ability to attenuate PMN recruitment. These observations support the notion that *Giardia* parasites may reduce host inflammatory responses. Such effects and their mechanisms have yet to be established *in vivo*. The present report demonstrates that *Giardia* infections attenuate granulocyte infiltration during acute experimental colitis induced by *Clostridrium difficile* toxins TcdA and TcdB, and markedly modulate the expression of several pro-inflammatory mediators, in an isolate-dependent manner. This study also shows that *Giardia* trophozoites decrease expression of a variety of inflammatory mediators when parasites are co-incubated with inflamed biopsy tissues from patients with Crohn’s disease. Collectively, these results indicate for the first time that certain *Giardia* isolates are capable of attenuating intestinal inflammatory responses in inflammatory settings *in vivo and ex vivo*.

## Materials and Methods

### Ethics statement

All studies involving human colonic mucosal biopsy tissues were approved by the Conjoint Health Research Ethics Board (CHREB) at the University of Calgary and the Calgary Health Region. In accordance with CHREB guidelines, adult subjects used in this study provided informed, written consent and a parent or guardian of any child participant provided informed, written consent on their behalf. Animal experiments were approved by the Life and Environmental Sciences Animal Care Committee of the University of Calgary, conducted in compliance with that approval, and followed guidelines established by the Canadian Council of Animal Care. All isolates used in this study have been used for over 15 years in the laboratory. Therefore, their isolation did not require their approval from an ethical review board at their time of collection.

### Human biopsy tissues

Adapted from previous protocols [41], [45], intestinal biopsy tissues were collected from the descending colon of patients with active Crohn’s disease (CD). Upon collection, samples were washed once in Dulbecco’s PBS (Sigma-Aldrich) containing 0.016% 1,4-Diothioerythritol (Sigma-Aldrich) to remove loosely adherent mucous and bacteria followed by three washes with phosphate-buffered saline (PBS). Following this, biopsy tissues were placed in 96-well plates and incubated in 300 µL of OptiMEM (Life Technologies) at 37°C, 5% CO_2_, and 96% humidity. All studies were performed in duplicate, whereby multiple biopsy tissues were collected from each patient.

### Parasites

*Giardia* NF trophozoites were originally obtained from a water sample during an outbreak of giardiasis in Newfoundland, Canada [46], while *Giardia* GS/M clone H7 trophozoites were obtained from ATCC (50581) as previously described [47]. Trophozoites were grown axenically in 15 mL polystyrene tubes (Becton-Dickinson Falcon) in Keister’s modified TYI-S-33 medium [48], [49] supplemented with piperacillin (Sigma-Aldrich) and used at peak density culture.

### *Giardia* trophozoite isolation

Confluent tubes of *Giardia* NF or GS/M trophozoites were harvested by cold shock on ice for 30 minutes, pooled into 50 mL polypropylene tubes (Falcon), and centrifuged at 500×*g* for 10 minutes. The resulting pellets were re-suspended in a total of 10 mL of ice cold PBS (Sigma-Aldrich) and centrifuged for 10 minutes at 500×*g*. Pellets were then re-suspended in 3 mL of fresh PBS, trophozoites were enumerated with a hemocytometer, and adjusted to the required concentration. For *ex vivo* human biopsy experiments, *Giardia* trophozoites were adjusted to a concentration of 5.0×10^6^ trophozoites/well, while trophozoites were adjusted to a concentration of 1.0×10^8^ trophozoites/mL for *in vivo* experiments. For *ex vivo* experiments, cells were co-incubated with *Giardia* trophozoites at 37°C, 5% CO_2_, and 96% humidity for 6 hours.

### Giardia in vivo infection

Using a previously described *Giardia* infection model [50], [51], male C57BL/6 mice aged 4 to 6 weeks (Charles River Laboratories, Sherbrooke, QC) were acclimatized for 1 week prior to infection with *Giardia* NF or GS/M trophozoites. Forty-eight hours prior to oral gavage, all mice were administered broad-spectrum antibiotics to their drinking water ad libitum (1.4 mg/mL neomycin (Alfa Aesar), 1.0 mg/mL ampicillin (Alfa Aesar) and 1.0 mg/mL vancomycin (Alfa Aesar)). This regimen was maintained for the entire duration of the study. After 48 hours, mice were administered 10^7^ *Giardia* NF or GS/M trophozoites in 0.1 mL of TYI-S-33 medium or 0.1 mL TYI-S-33 medium by oral gavage. After 7 days, small intestinal parasite loads were quantified. Mice were euthanized and the first 3 cm of the small intestine distal to the ligament of Treitz were opened longitudinally, placed in 1.5 mL Eppendorf tubes containing 1 mL of PBS, and kept on ice for 15 minutes. After 15-minute incubation, tubes were vortexed, and trophozoite numbers were visually enumerated under light microscope, and total concentration was extrapolated using a hemocytometer.

### Clostridium difficile TcdAB-induced colitis

Following a previously described model of *C. difficile* toxin-induced colitis [52], mice infected with *Giardia* NF or GS/M trophozoites for 7 days were intra-rectally (i.r.) administered a 100 µg solution of *C. difficile* A/B toxin (TcdA/TcdB) for 3 hours. Briefly, a 5F infant feeding tube catheter containing side ports (Mallinckrodt Inc.) was lubricated with a water-soluble personal lubricant and inserted 2.5 cm into the colon. With pressure applied to the anal area to prevent leakage, 100 µL of a 1.0 µg/µL TcdA/TcdB solution diluted in PBS was slowly administered over a period of 30 seconds. Following this, the tube was slowly removed and pressure was maintained for another 30 seconds. Vehicle control-treated control groups were administered PBS.

### Tissue collection

Mice were euthanized via cervical dislocation 3 hours post intracolonic instillation of TcdAB or PBS. The colon was removed and samples were collected for bead-based cytokine analysis, myeloperoxidase (MPO) assays, or fixed in a fresh 4% paraformaldehyde solution for immunofluorescence, as described below. Colonic tissue samples collected for histology and immunohistochemistry were fixed in 4% paraformaldehyde solution overnight at 4°C, and, subsequently, washed and transferred to PBS at 4°C.

### Cytokine analysis

Supernatants from *ex vivo* human biopsy tissue experiments were collected and centrifuged at 10,000 *g* for 15 minutes at 4°C. Resulting supernatants were decanted and stored at −70°C. Colonic tissue samples and biopsy tissues were weighed and collected in 2 mL Fast-Prep tubes (MP Biomedicals) containing a mixture of 0.9–2.0 mm stainless steel beads (NextAdvance). Tissue samples were suspended in lysis buffer [20 mM Tris-HCl (pH 7.5), 150 mM NaCl, 0.5% Tween-20 and a Complete Minitab protease inhibitor cocktail (Roche)] at a ratio of 50 mg tissue per 1 mL lysis buffer and homogenized using a Fast-Prep24 device (MP Biochemicals) at speed 6.0 for 40 seconds. The resulting homogenate solution was collected into pyrogen-free 1.5 mL Eppendorf tubes and centrifuged at 10,000 *g* for 15 minutes at 4°C. Supernatants and tissue homogenate cytokine levels were assessed using a Luminex XMap assay according to manufacturer’s instructions (Luminex Corp.).

### Tissue myeloperoxidase assay

Myeloperoxidase (MPO) activity was used as a marker of granulocyte infiltration [53]. Colonic tissue samples were weighed and collected in 2 mL Fast-Prep tubes (MP Biomedicals) containing a mixture of 0.9–2.0 mm stainless steel beads (NextAdvance). Tissue samples were suspended in 50 mM potassium phosphate buffer containing 5 mg/mL hexadecyltrimethylammonium bromide (Sigma-Aldrich) at a ratio of 50 mg tissue per 1 mL lysis buffer. Samples were then homogenized using a Fast-Prep24 device (MP Biochemicals) at speed 6.0 for 40 seconds. The resulting homogenate solution was collected into pyrogen-free 1.5 mL Eppendorf tubes and centrifuged at 10,000×*g* for 15 minutes at 4°C. Seven µL of the resulting supernatant was added to a standard 96-well plate along with 200 µL of the reaction mixture (comprised of 0.005 g O-dianisidine (Sigma-Aldrich), 30 mL of distilled H_2_O, 3.33 mL of potassium phosphate buffer, and 17 µL of 1% H_2_O_2_). Using a microplate scanner (SpectraMax M2^e^, Molecular Devices, Sunnyvale, CA), three absorbance readings at 450 nm were recorded every 30 seconds. MPO activity was measured as units of activity per milligram of tissue, with 1 unit of MPO being defined as the amount required to degrade 1 µmol of H_2_O_2_ per minute at room temperature.

### Immunohistochemistry

Samples were dehydrated and embedded in paraffin wax, cut on a cryostat into 8 µm sections, and mounted on poly-D-lysine coated slides. Slides were deparaffinised and incubated in antigen retrieval solution (Tris/EDTA, pH 9.0 solution for 30 minutes at 95 degrees C. Slides were then blocked in tissue blocking buffer (1% BSA, 0.1% Triton X-100 in PBS) for 1 hour (3×20 minute washes) and incubated with an anti-MPO antibody (abcam ab9535) (1 in 200 dilution) overnight at 4°C. At room temperature, slides were washed for 1 hour (3×20 minutes), incubated for 1 hour with secondary antibody (1 in 1000), and then washed for 1 hour (3×20 minutes). After this, slides were mounted with Fluoroshield™ containing DAPI (Sigma-Aldrich) and visualized using a Leica DMR fluorescence microscope at 40× magnification.

### Statistics

Parametric data are expressed as means ± standard error of measurement, while non-parametric data is expressed as a dotplots with median and range. All statistical analyses were performed using GraphPad Prism 6 software, and normality of the data was assessed prior to analysis. Parametric comparisons were made using one-way ANOVA with Tukey’s post hoc analysis. Non-parametric comparisons were made using a Mann Whitney test. Statistical significance was established at p<0.05 (*).

## Results

### *Giardia* infections attenuate granulocyte infiltration during TcdAB-induced colitis in an isolate-dependent manner

**Figure 1:**
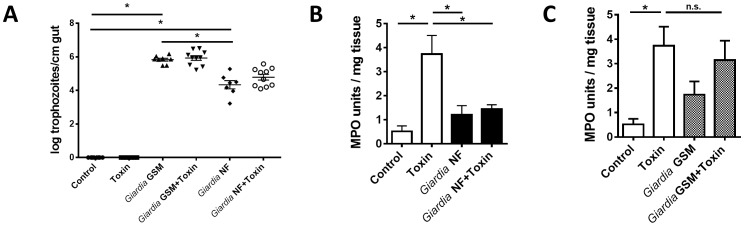
*Giardia* NF infections attenuate granulocyte recruitment in a model of *C. difficile* TcdAB-induced colitis. Male C57BL/6 mice aged 4 to 6 weeks were infected with *Giardia* NF or GS/M trophozoites. 7 days post-infection, animals were given 100 µg of TcdAB or PBS i.r. and, after 3 hours, animals were euthanized. (A) Trophozoites counts within the upper 3 cm of the jejunum were determined. (B) Colonic myeloperoxidase (MPO) activity was determined between uninfected controls and *Giardia* NF-infected animals given 100 µg TcdAB or PBS. (C) Colonic MPO activity was determined between uninfected controls and *Giardia* GS/M-infected animals given 100 µg TcdAB or PBS. (D) Values were determined via bead-based cytokine assay. All data are representative of two independent experiments (n = 5–11/group) and represented as mean ± SEM. * p<0.05.

Experiments were performed to determine whether *in vivo* assemblage A *Giardia* NF or assemblage B *Giardia* GS/M infections were capable of attenuating acute intestinal inflammatory responses to intracolonic administration of *C. difficile* toxin A (TcdA) and toxin B (TcdB). This model of intestinal inflammation was selected because it induces a rapid, acute intestinal inflammatory response resulting from the administration of bacterial toxins, and *Giardia* infections have been reported to occur concurrently with *C. difficile* [16]. Small intestinal trophozoites numbers in *Giardia* GS/M-infected animals were higher than numbers from NF-infected animals (Figure 1A). These results are consistent with previous observations that assemblage B *Giardia* isolates, such as GS/M, have higher parasite burdens compared to assemblage A isolates, such as NF trophozoites [50], [54]. Administration of *C. difficile* TcdAB for 3 hours did not change trophozoite numbers (Figure 1A).

**Figure 2:**
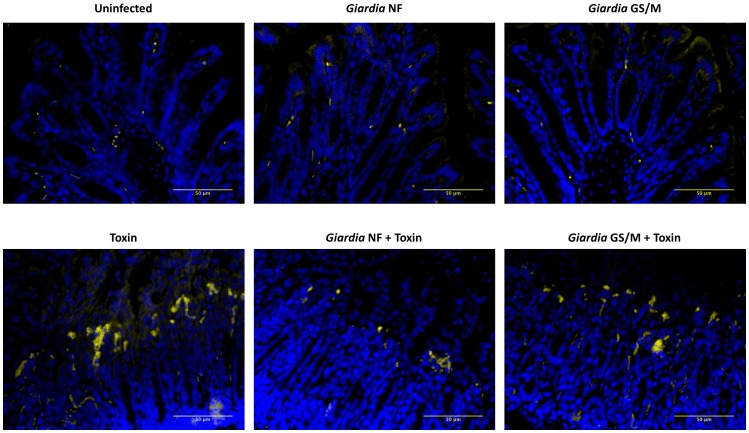
*Giardia* NF-infected animals exhibit reduced MPO-positive cells following administration of *C. difficile* TcdAB. Male C57BL/6 mice aged 4 to 6 weeks were infected with *Giardia* NF or GS/M trophozoites. 7 days post-infection, animals were given 100 µg of TcdAB or PBS i.r. and, after 3 hours, animals were euthanized. Representative immunohistochemical images of MPO-positive cells (yellow) counterstained with DAPI (blue) in colonic tissues of uninfected animals or animals infected with *Giardia* NF or GS/M trophozoites following i.r. instillation of 100 µg TcdAB or PBS. All data are representative of two independent experiments (n = 5–11/group) and taken at the same magnification (400×). Bars equal 50 µm for all micrographs.

As previously demonstrated [52], intracolonic administration of 100 µg of *C. difficile* TcdAB significantly increased colonic tissue MPO activity (Figure 1B and C). As determined via immunofluorescence, numbers of MPO-positive cells increased in colonic tissues of TdcAB-trated animals compared to control animals (Figure 2). In *Giardia* NF-infected animals administered intracolonically with 100 µg of TcdAB, colonic MPO activity (Figure 1B) and numbers of MPO-positive cells were lower when compared against control animals (Figure 2). Contrastingly, MPO activity levels (Figure 1C) and numbers of MPO-positive cells (Figure 2) in *Giardia* GS/M-infected animals intracolonically instilled with 100**µg of TcdAB were not significantly different from TcdAB-administered controls.

### *Giardia* NF infections attenuate neutrophil-associated cytokines during TcdAB-induced colitis

**Figure 3:**
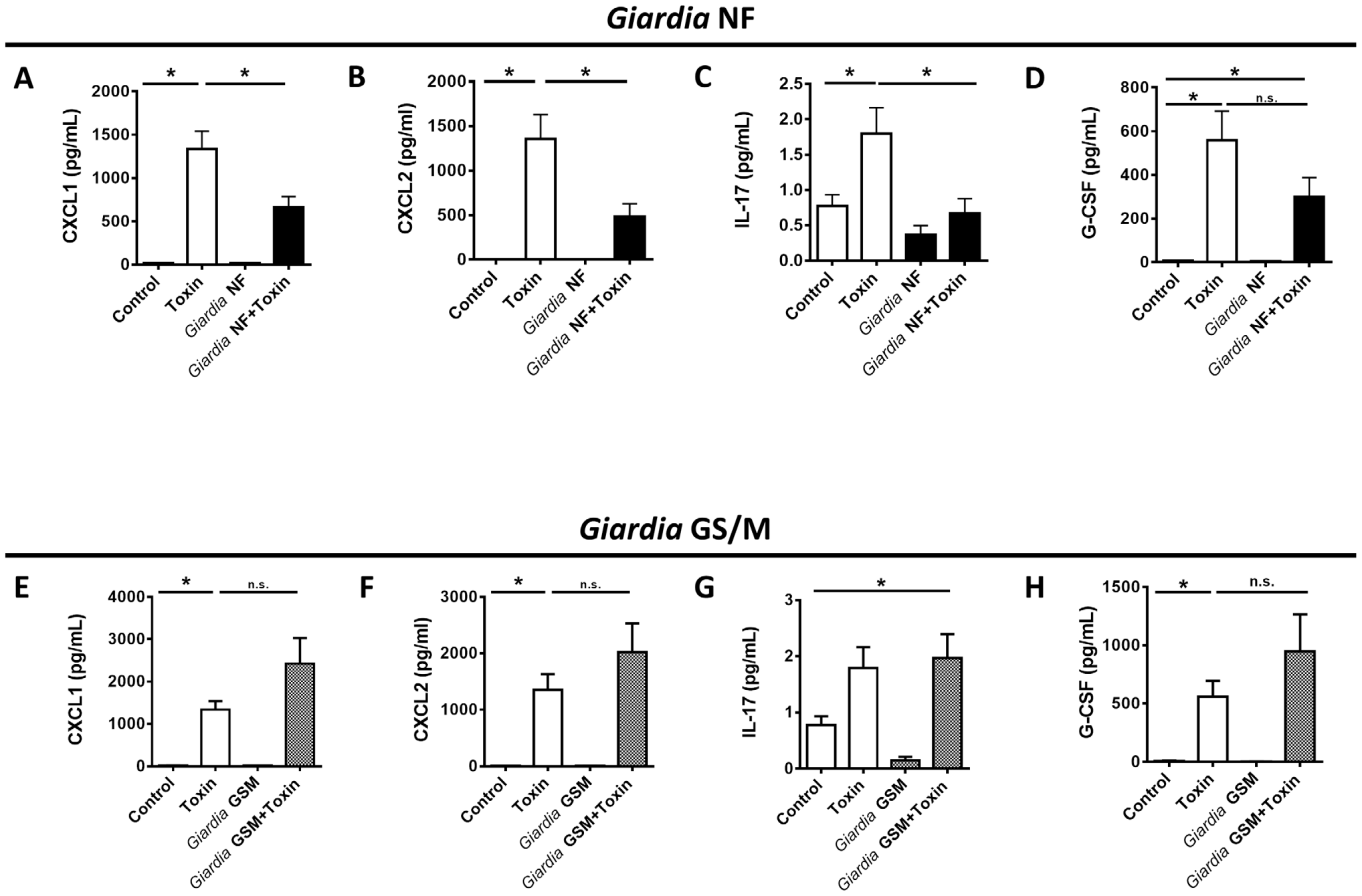
*Giardia* NF infections attenuate colonic expression levels of several PMN-associated mediators following administration of *C. difficile* TcdAB. Male C57BL/6 mice, aged 4 to 6 weeks, were infected with *Giardia* NF or GS/M trophozoites. 7 days post-infection, animals were given 100 µg of TcdAB or PBS i.r. and, after 3 hours, animals were euthanized. Levels of (A) CXCL1, (B) CXCL2, (C) IL-17, and (D) G-CSF were compared between *Giardia* NF-infected animals and uninfected animals given i.r. 100 µg of TcdAB or PBS. Levels of (E) CXCL1, (F) CXCL2, (G) IL-17, and (H) G-CSF were compared between *Giardia* GS/M-infected animals and uninfected animals given i.r. 100 µg of TcdAB or PBS. Values were determined via bead-based cytokine assay. All data are representative of two independent experiments (n = 5–11/group) and represented as mean ± SEM. n.s. = not significant * p<0.05.

A bead-based cytokine assay on colonic tissue samples was performed to determine whether *Giardia* NF infections attenuated protein levels of pro-inflammatory cytokines and chemokines associated with granulocyte tissue recruitment. Compared to control animals, intracolonic instillation of 100 µg of TcdAB significantly increased tissue levels of several PMN-associated mediators, including CXCL1, CXCL2, IL-17 and G-CSF (Figure 3). Colonic tissues collected from *Giardia* NF infected-animals administered TcdAB. demonstrated significantly lower protein levels of CXCL1, CXCL2, and IL-17 (Figure 3A to C), while G-CSF protein levels were similar from uninfected TcdAB-treated animals (Figure 3D). Notably, colonic protein levels of CXCL1, CXCL2, IL-17, and G-CSF in *Giardia* GS/M-infected animals were similar from uninfected TcdAB-treated animals (Figure 3E to H). These results support above observations that granulocyte infiltration is not attenuated in *Giardia* GS/M infected animals instilled with TcdAB, and suggest attenuation of granulocyte infiltration in *Giardia* NF-infected animals given TcdAB may result from decreased expression of PMN-associated mediators. These results also indicate that *Giardia*-mediated attenuation of PMN-associated mediators induced via TcdAB occurs in an isolate-dependent manner.

### *Giardia* GSM reduce colonic expression of several pro-inflammatory cytokines during TcdAB-induced colitis

**Figure 4:**
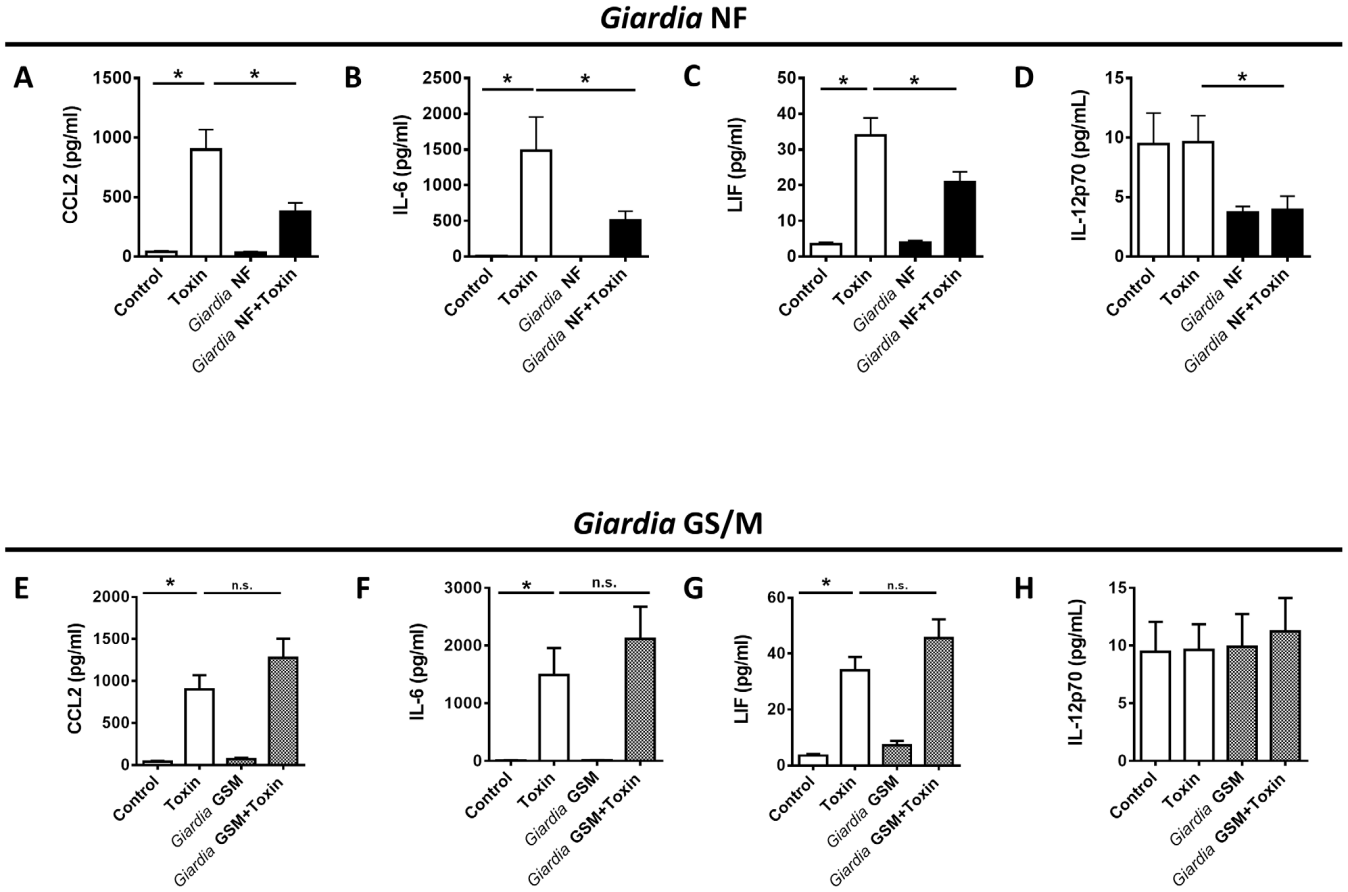
*Giardia* NF infections attenuate colonic expression levels of other pro-inflammatory mediators following administration of *C. difficile* TcdAB. Male C57BL/6 mice, aged 4 to 6 weeks, were infected with *Giardia* NF or GS/M trophozoites. 7 days post-infection, animals were given 100 µg of TcdAB or PBS i.r. and, after 3 hours, animals were euthanized. Levels of (A) CCL2, (B) IL-6, (C) LIF, and (D) IL-12p70 were compared between *Giardia* NF-infected animals and uninfected animals given i.r. 100 µg of TcdAB or PBS. Levels of (E) CCL2, (F) IL-6, (G) LIF, and (H) IL-12p70 were compared between *Giardia* GS/M-infected animals and uninfected animals given i.r. 100 µg of TcdAB or PBS. Values were determined via bead-based cytokine assay. All data are representative of two independent experiments (n = 5–11/group) and represented as mean ± SEM. n.s. = not significant * p<0.05.

After focusing on neutrophil-released cytokines, we investigated whether *Giardia* infections attenuated colonic expression of other pro-inflammatory cytokines. Compared to uninfected animals given PBS, intracolonic instillation of TcdAB to uninfected animals also resulted in heightened protein levels of CCL2, IL-6, and leukocyte inhibitory factor (LIF) (Figure 4) and IL-1β, IL-5, CXCL10, and CCL11 (Figure S1). Colonic protein levels of CCL2, IL-6, and LIF were significantly reduced in *Giardia* NF-infected animals given intracolonically with TcdAB (Figure 4A to C). In addition, colonic IL-12p70 levels were significantly greater in uninfected TcdAB-treated controls when compared to *Giardia* NF infected animals treated with TcdAB (Figure 4D). Conversely, CCL2, IL-6, LIF, and IL-12p70 levels in *Giardia* GS/M-infected animals treated with TcdAB were not significantly different from animals given TcdAB without giardiasis (Figure 4E to H). These results demonstrate that *G. duodenalis* NF attenuate colonic levels of CCL2, IL-6, LIF, and, potentially, IL-12p70 induced by 100 µg intracolonically of TcdAB in an isolate-dependent manner. *Giardia* NF-infections was unable to attenuate colonic expression levels of IL-1β, IL-5, CXCL10, and CCL11 (Fig. S1) Protein levels of IL-1α, IL-7, IL-9, IL-10, IL-12p40, IL-15, IFN-γ, CCL3, CCL4, CXCL9, VEGF remained unchanged between all animal groups (Figure S2). Therefore, following TcdAB instillation *Giardia* NF specifically targeted and decreased colonic expression of pro-inflammatory cytokines that recruit PMNs (CXCL1, CXCL2, and IL-17) and other cytokines (CCL2, IL-6, and LIF).

**Figure 5:**
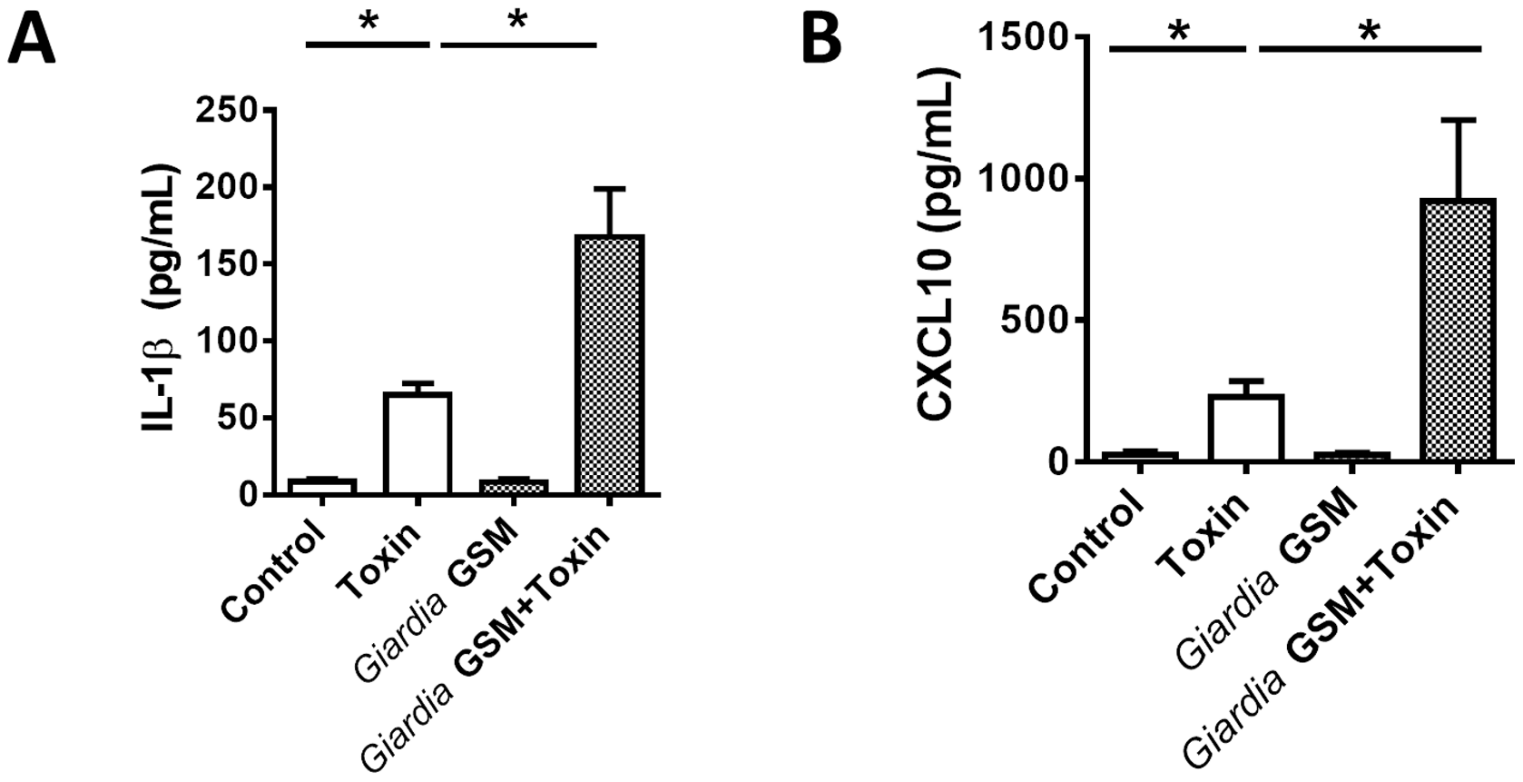
*Giardia* GS/M infections increase colonic expression levels of IL-1β and CXCL10 following administration of *C. difficile* TcdAB. Male C57BL/6 mice, aged 4 to 6 weeks, were infected with *Giardia* GS/M trophozoites. 7 days post-infection, animals were given 100 µg of TcdAB or PBS i.r. and, after 3 hours, animals were euthanized. Levels of (A) IL-1β and (B) CXCL10 were compared between *Giardia* GS/M-infected animals and uninfected animals given i.r. 100 µg of TcdAB or PBS. Values were determined via bead-based cytokine assay. All data are representative of two independent experiments (n = 5–11/group) and represented as mean ± SEM. * p<0.05.

Bead-based cytokine analysis of colonic tissues indicated *Giardia* GS/M infections upregulated colonic expression levels of a subset of pro-inflammatory mediators, following TcdAB. In uninfected animals, colonic administration of TcdAB significantly increased colonic expression levels of IL-1β and CXCL10 (Figure 5), and protein levels of these mediators were significantly increased in *Giardia* GS/M-infected animals compared to uninfected TcdAB-treated animals (Figure 5). *Giardia* GS/M*-*infected animals given TcdAB also demonstrated increased colonic protein levels of several inflammatory mediators not initially increased via colonic instillation of TcdAB (Figure S3). This did not appear to be a global increase in expression of inflammatory mediators, as CCL3 and CCL11 concentrations were similar between *Giardia* GS/M-infected animals given TcdAB and uninfected TcdAB controls (Figure S4). Several inflammatory mediators also remained unchanged between all experimental groups (Figure S5). These results suggest *Giardia* GS/M infections enhance expression of pro-inflammatory cytokines following colonic instillation of TcdAB. These data indicate that *Giardia-*infections can enhance or decrease the expression of inflammatory mediators following administration of a bacterial toxin, events which are isolate-specific.

### *Giardia* NF trophozoites attenuate various inflammatory mediators from inflamed human mucosal biopsy tissues *ex vivo*

**Figure 6:**
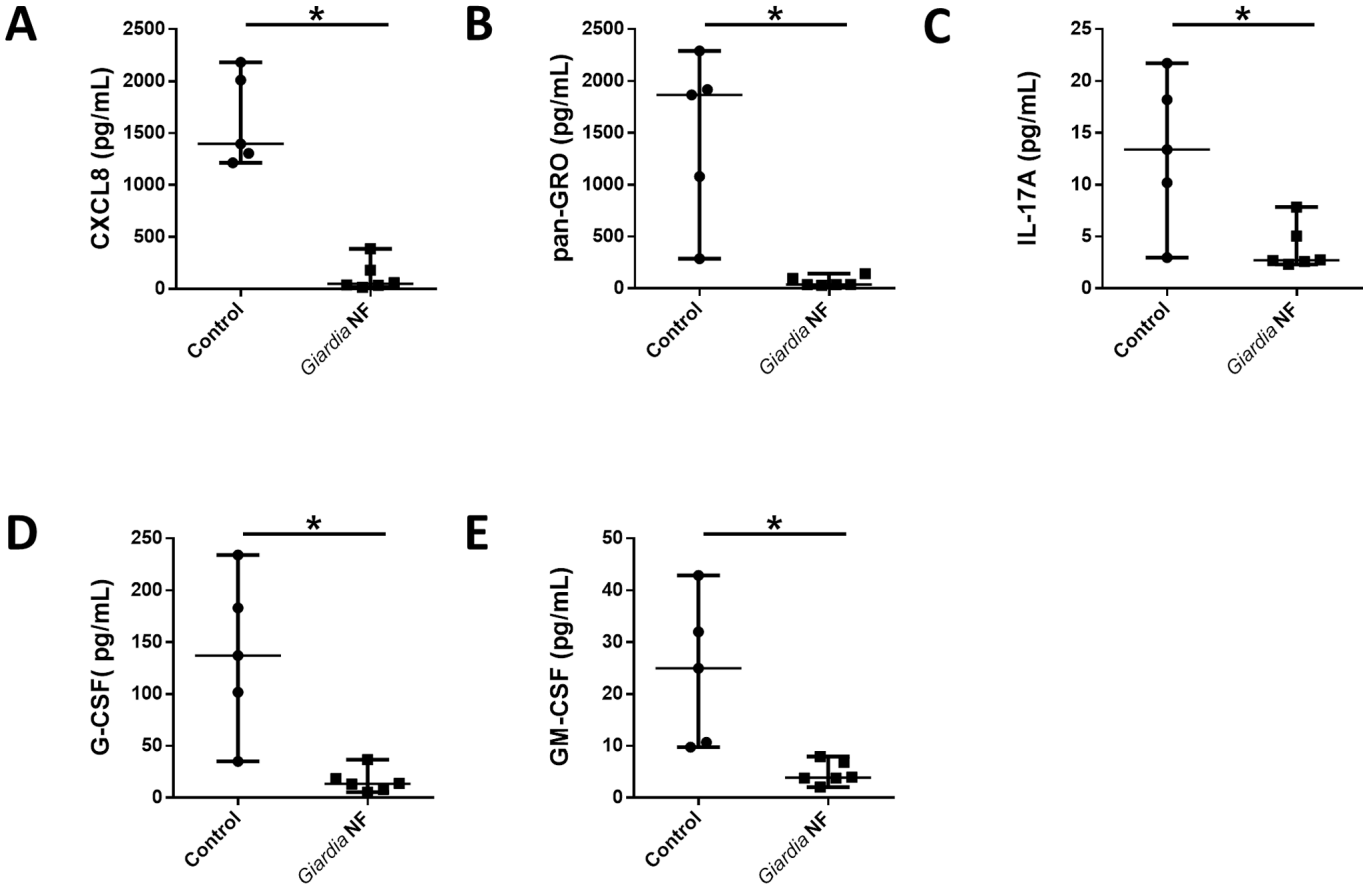
*Giardia* NF trophozoites attenuate several PMN-associated mediators released from human colonic mucosal biopsies. Human descending colon mucosal biopsy tissues obtained from areas of active inflammation from patients with active Crohn’s disease (CD) were incubated with 5.0×10^6^ *Giardia* NF trophozoites for 6 hours. Supernatant levels of (A) IL-8/CXCL8, (B) GRO, (C) CCL3, (D) IL-17A, (E) G-CSF, and (F) GM-CSF were determined via a bead-based cytokine assay. All data are representative of three independent experiments (n = 4–6/group) and represented as median with the range. * p<0.05.

**Figure 7:**
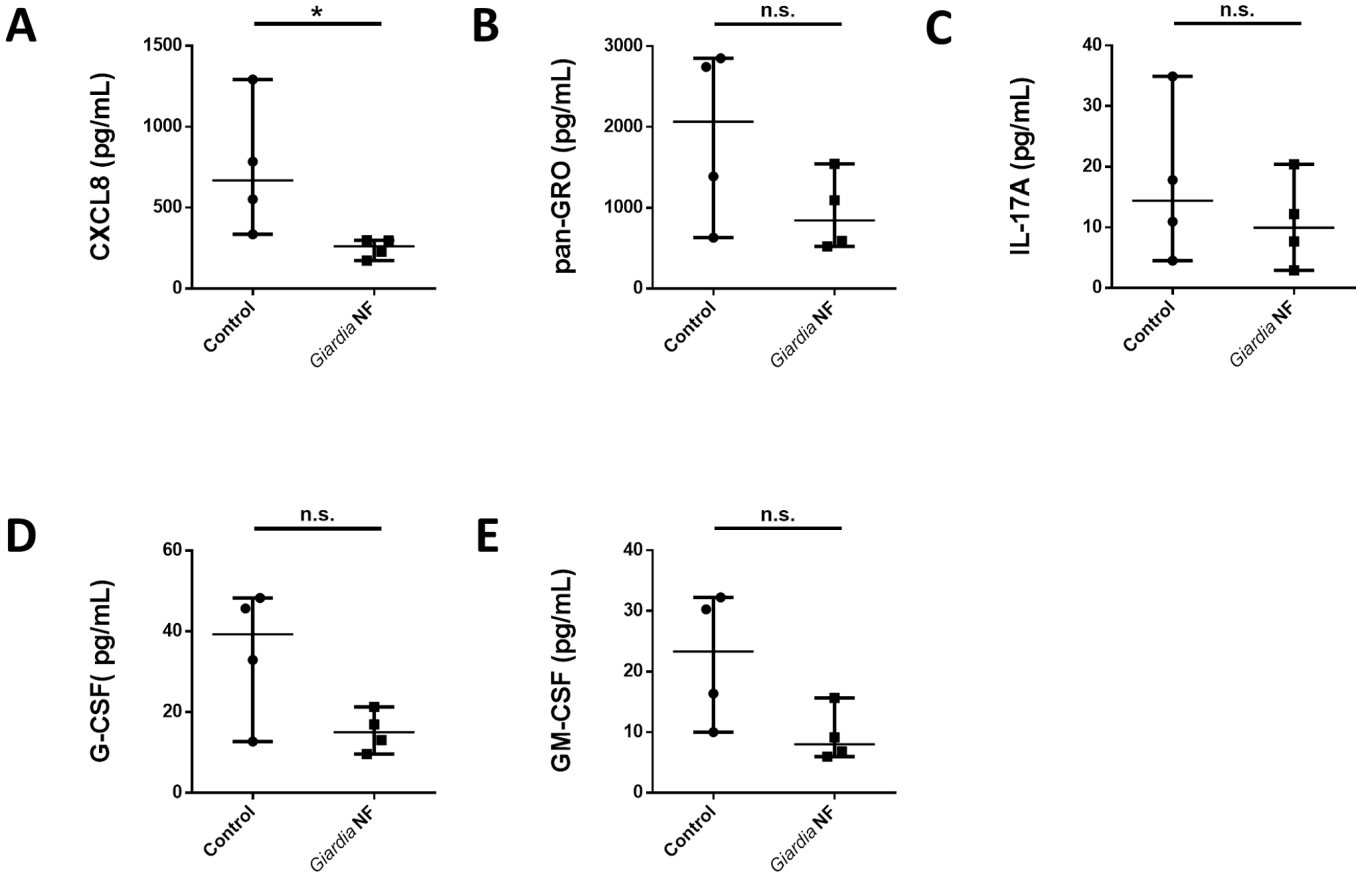
*Giardia* NF trophozoites attenuate the expression of IL-8/CXCL8 assessed in colonic mucosal biopsy homogenates. Human descending colon mucosal biopsy tissues obtained from areas of active inflammation from patients with active Crohn’s disease (CD) were incubated with 5.0×10^6^ *Giardia* NF trophozoites for 6 hours. Tissue homogenate levels of (A) CXCL8, (B) GRO, (C) CCL3, (D) IL-17A, (E) G-CSF, and (F) GM-CSF were determined via a bead-based cytokine assay. All data are representative of three independent experiments (n = 4–6/group) and represented as median with the range. * p<0.05.

Our above results demonstrated *in vivo* that *Giardia* NF attenuated PMN accumulation and expression of pro-inflammatory cytokines following colonic administration of TcdA/TcdB. Therefore, we next assessed whether this same isolate was capable of attenuating similar inflammatory mediators from inflamed human colonic tissue. Using a previously published model [41], *Giardia* NF trophozoites were co-incubated *ex vivo* with colonic mucosal biopsy tissues collected the descending colon of patients with active CD, where supernatants and biopsy tissue homogenates were analyzed via a bead-based cytokine assay. Supernatants collected from co-incubation of *Giardia* NF trophozoites with inflamed biopsy tissues displayed significantly reduced supernatant levels of cytokines associated with granulocyte recruitment, including IL-8/CXCL8, growth-related oncogene (GRO) family proteins (CXCL1-3), IL-17A, G-CSF, and GM-CSF (Figure 6). Co-incubation of *Giardia* NF trophozoites with biopsy tissues also resulted in decreased IL-8/CXCL8 levels detected within biopsy tissue homogenates (Figure 7A), but not G-CSF and GM-CSF.

**Figure 8:**
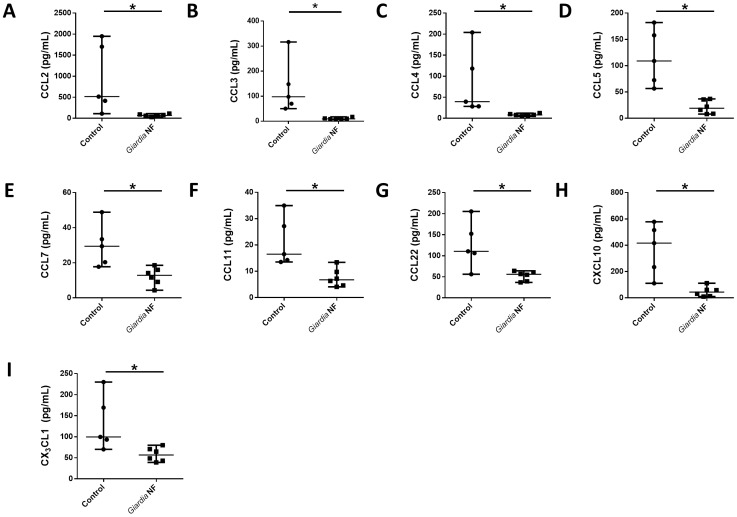
*Giardia* NF trophozoites attenuate the release of several chemokines from colonic mucosal biopsies. Human descending colon mucosal biopsy tissues obtained from areas of active inflammation from patients with active Crohn’s disease (CD) were incubated with 5.0×10^6^ *Giardia* NF trophozoites for 6 hours. Supernatant levels of (A) CCL2, (B) CCL4, (C) CCL5, (D) CCL7, (E) CCL11, (F) CCL22, (G) CXCL10, and (H) CX_3_CL1 were determined via a bead-based cytokine assay. All data are representative of three independent experiments (n = 4–6/group) and represented as median with the range. * p<0.05.

**Figure 9:**
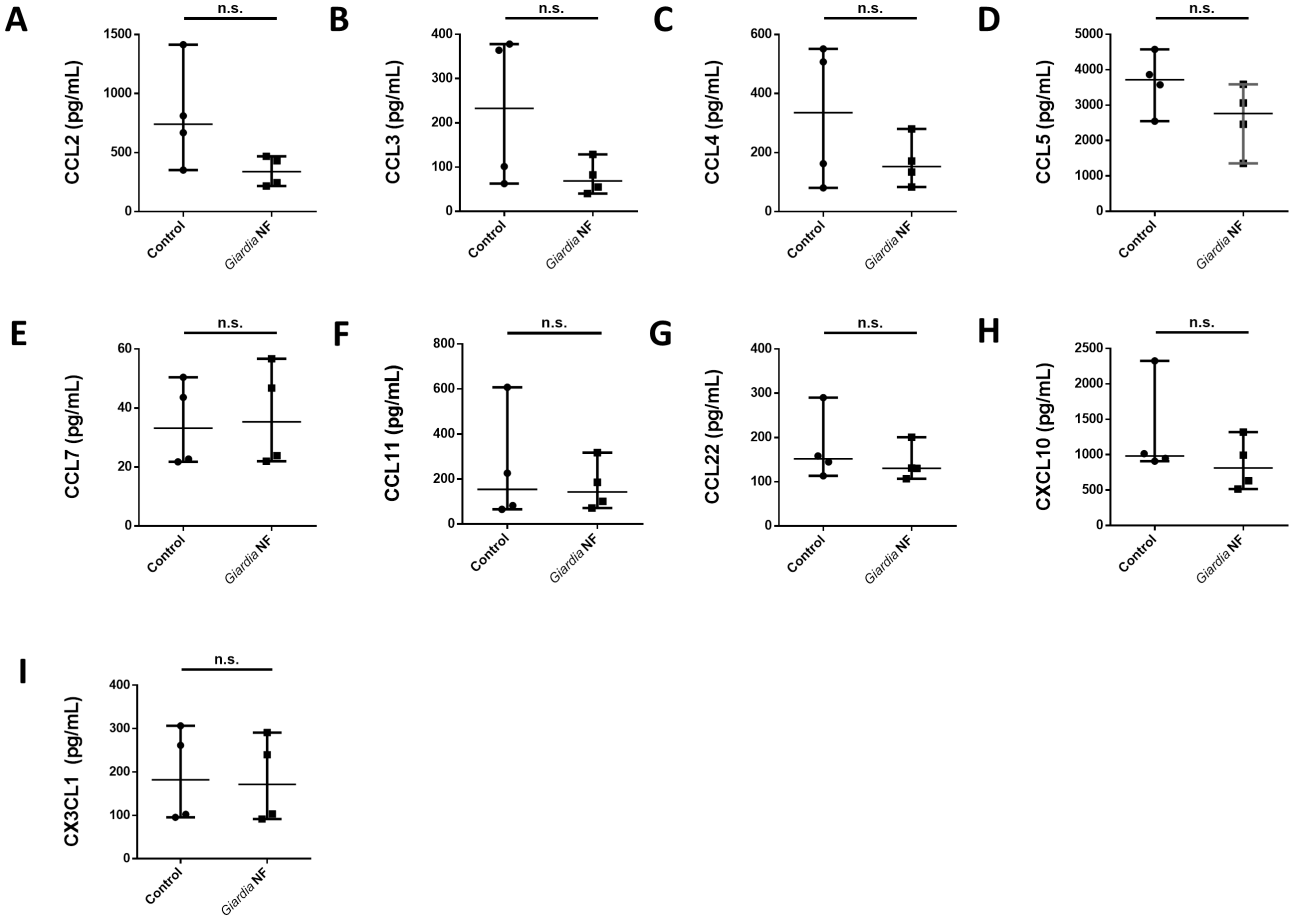
*Giardia* NF trophozoites do not attenuate the expression of several chemokines assessed in colonic mucosal biopsy tissue homogenates. Human descending colon mucosal biopsy tissues obtained from areas of active inflammation from patients with active Crohn’s disease (CD) were incubated with 5.0×10^6^ *Giardia* NF trophozoites for 6 hours. Tissue homogenate levels of (A) CCL2, (B) CCL4, (C) CCL5, (D) CCL7, (E) CCL11, (F) CCL22, (G) CXCL10, and (H) CX_3_CL1 were determined via a bead-based cytokine assay. All data are representative of three independent experiments (n = 4–6/group) and represented as median with the range. * p<0.05.

**Figure 10:**
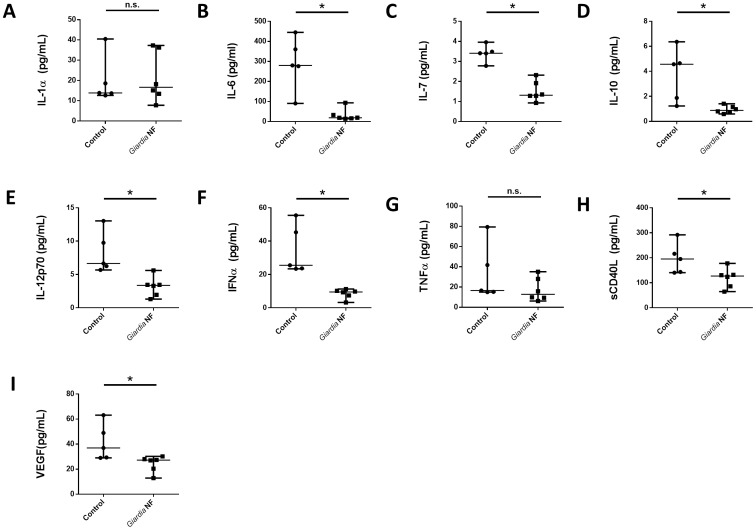
*Giardia* NF trophozoites attenuate the release of several pro-inflammatory mediators from colonic mucosal biopsies. Human descending colon mucosal biopsy tissues obtained from areas of active inflammation from patients with active Crohn’s disease (CD) were incubated with 5.0×10^6^ *Giardia* NF trophozoites for 6 hours. Supernatant levels of (A) IL-1α, (B) IL-6, (C) IL-7, (D) IL-10, (E) IL-12p70, (F) IFNα, (G) TNFα, (H) sCD40L, and (I) VEGF were determined via a bead-based cytokine assay. All data are representative of three independent experiments (n = 4–6/group) and represented as median with the range. * p<0.05.

**Figure 11:**
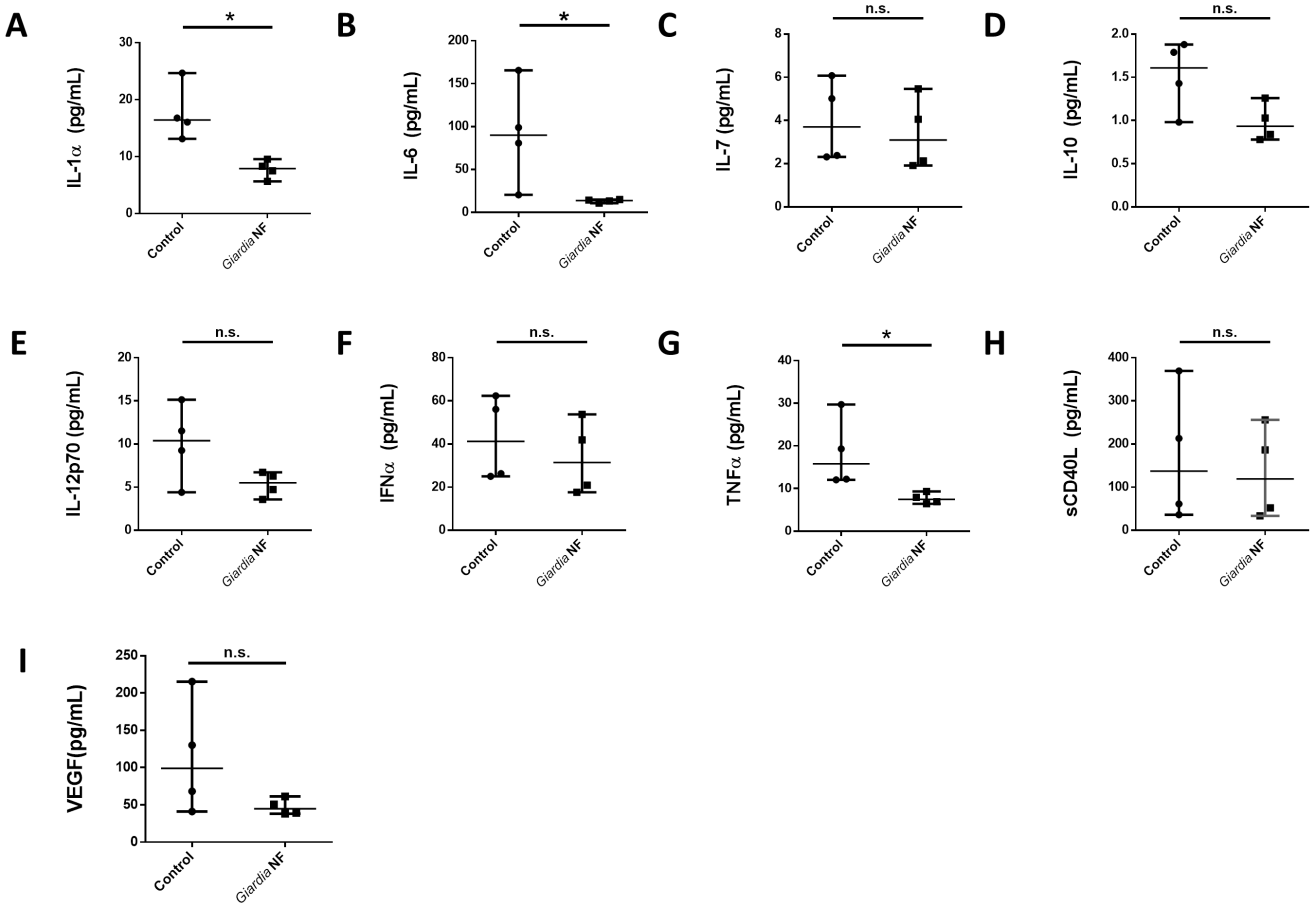
*Giardia* NF trophozoites attenuate the expression of certain pro-inflammatory mediators assessed in colonic mucosal biopsy tissue homogenates. Human descending colon mucosal biopsy tissues obtained from areas of active inflammation from patients with active Crohn’s disease (CD) were incubated with 5.0×10^6^ *Giardia* NF trophozoites for 6 hours. Tissue homogenate levels of (A) IL-1α, (B) IL-6, (C) IL-7, (D) IL-10, (E) IL-12p70, (F) IFNα, (G) TNFα, (H) sCD40L, and (I) VEGF were determined via a bead-based cytokine assay. All data are representative of three independent experiments (n = 4–6/group) and represented as median with the range. * p<0.05.

As *in vivo Giardia* NF attenuated colonic levels of CCL2, IL-6, LIF, and, potentially, IL-12p70 (Figure 4), we investigated whether live trophozoites were capable of attenuating protein expression of other chemokines. Supernatant levels of CCL2-5, CCL7, CCL11, CCL22, CXCL10, and CX_3_CL1 were significantly reduced in biopsy tissues co-incubated with *Giardia* NF trophozoites compared to control biopsy tissues (Figure 8). Tissue homogenate levels of these mediators were similar when biopsy tissues were incubated in the presence or absence of *Giardia* NF trophozoites (Figure 9). Therefore, attenuation of these mediators by *G. duodenalis* NF trophozoites appears to occur following their release into supernatants. These results also suggest that *Giardia* NF trophozoites are capable of attenuating CCL2 in different experimental models of inflammation. Compared to control biopsy supernatant, co-incubation of *Giardia* NF trophozoites with inflamed biopsy tissues *ex vivo* resulted in significant attenuation of IL-6, IL-7, IL-10, IL-12p70, IFN-α, soluble CD40 ligand (sCD40L) and vascular endothelial growth factor (VEGF) (Figure 10). Tissue homogenate levels of IL-1α, IL-6, and TNF-α were significantly reduced in biopsy tissue homogenates co-incubated with *Giardia* NF trophozoites, compared to biopsy tissue homogenates incubated in the absence of *Giardia* NF trophozoites (Figure 11). These results suggest that *Giardia* NF trophozoites attenuate secreted IL-7, IL-10, IL-12p70, IFN-α, sCD40L, and VEGF from inflamed mucosal biopsy tissues *ex vivo*, while also attenuating tissue levels of IL-1α, IL-6, and TNF-α. These results also suggest that *Giardia* infections are capable of attenuating IL-6 and IL-12p70 in during an *in vivo* infection and following incubation with human intestinal biopsy tissues *ex vivo*. Our data also indicated that *Giardia* NF trophozoites did not attenuate the expression of pro-inflammatory cytokines (Figure S6 and S7) or growth factors (Figure S8 and S9) within supernatants or biopsy tissue homogenates.

## Discussion

Results from the present study demonstrate novel mechanisms through which certain *Giardia* infections were able to attenuate pro-inflammatory responses elicited by an inflammatory bacterial toxin *in vivo,* or from inflamed human intestinal tissues *ex vivo.* Attenuation of granulocyte infiltration in *Giardia* NF infected animals given TcdAB occurred concomitantly with reduced colonic expression of several inflammatory mediators, including those associated with granulocyte tissue recruitment. Several of these factors were also decreased following co-incubation of *Giardia* NF trophozoites and *ex vivo* inflamed human colonic mucosal biopsy tissues, and, therefore, support our *in vivo* observations that *Giardia* NF trophozoites are capable of attenuating factors associated with PMN recruitment. These results also reinforce previous observations that *Giardia* trophozoites are capable of attenuating IL-8/CXCL8 production from intestinal tissues *in vitro* and *ex vivo* [41]. Furthermore, our data indicate that *Giardia* trophozoites can attenuate the production of additional inflammatory mediators from intestinal tissues of differing origin, strengthening the notion that infection with specific *Giardia* isolates may modulate host immune responses in the gut. Interestingly *Giardia* GS/M-infected animals did not display reduced granulocyte infiltration nor decreased expression of inflammatory mediators. On the contrary, *Giardia* GS/M infections enhanced the expression of several factors initially upregulated following TcdAB administration and increased the expression of several factors not initially induced by TcdAB exposure. Therefore, our data suggest that *Giardia* infections attenuate intestinal pro-inflammatory responses in an isolate-dependent manner, and attenuation of such responses is associated with reduced tissue granulocyte infiltration. The results show that *Giardia* NF attenuate a variety of pro-inflammatory mediators when co-incubated with inflamed colonic mucosal biopsy tissues *ex vivo*, but the inability to decrease expression of all mediators examined suggests this process may be targeted towards certain inflammatory mediators. Moreover, observations that some inflammatory mediators are preferentially degraded within supernatants while others within tissues suggests that *Giardia* NF trophozoites possess multiple mechanisms capable of attenuating expression of inflammatory mediators in inflamed intestinal mucosal biopsy tissues.

Excessive PMN infiltration and activation contribute to several gastrointestinal inflammatory disorders, including the inflammatory bowel diseases (IBD) and *C. difficile* colitis [55]–[57]. The present findings indicate that *Giardia* NF infections *in vivo* attenuate PMN tissue recruitment induced by a pro-inflammatory bacterial toxin by reducing tissue expression of mediators that can contribute to the tissue recruitment of PMNs. Release of ELR+ chemokines, such as CXCL1, CXCL2, and IL-8/CXCL8, from a variety of intestinal tissue-resident cells promotes PMN accumulation and chemotaxis through intestinal tissues. In mice, these chemokines include CXCL1 and CXCL2, while in humans this also includes IL-8/CXCL8 [58]–[61]. Separately, enhanced expression of G-CSF promotes the release of PMNs into the bloodstream [31], [62], in a process that can be initiated via IL-17 production [32], [33]. Our results suggest that *Giardia* NF infections attenuate granulocyte infiltration into colonic tissues following colonic. TcdA/B by reducing colonic expression levels of CXCL1, CXCL2, and IL-17; these results are supported by observations that *Giardia* GS/M infections failed to attenuate colonic granulocyte infiltration and expression of PMN-associated mediators. In addition, *Giardia* NF parasites attenuated supernatant levels of IL-8/CXCL8, GRO-family proteins (CXCL1-3), IL-17, G-CSF, and GM-CSF when co-incubated with *ex vivo* descending colon mucosal biopsy tissues. Together, these data show for the first time that *Giardia* infections attenuate the release of a variety of mediators associated with PMN recruitment in different experimental models of inflammation, in an isolate-dependent manner.

Further research is needed to demonstrate whether the results from this study explain how *Giardia* infections can, in a strain-dependent manner, modulate the incidence of diarrheal disease [20], [22], and create an environment that is favourable for colonization by other gastrointestinal pathogens [18]. Indeed, the data suggest that *Giardia* may attenuate pro-inflammatory responses elicited by other co-infecting pathogens that can cause diarrheal disease, while other strains may enhance its development. Following their recruitment to tissues, PMNs are known to induce multiple pathophysiological events within intestinal tissues capable of causing diarrhea [28], [42]–[44]. Therefore, *Giardia* NF infections may protect against the development of diarrheal disease by attenuating PMN recruitment. *Giardia* GS/M infections did not enhance granulocyte infiltration following colonic of TcdAB; however, these infections resulted in enhanced expression of several pro-inflammatory mediators that could, potentially, enhance the development of diarrheal disease via other pathways. For example, IL-1β and IFNγ have been shown to modulate intestinal barrier dysfunction and ion secretion, and, resultantly, cause diarrhea [63]–[68]. As PMNs are essential to host defence against a variety of pathogens, more research is needed to determine whether attenuation of PMN recruitment by *Giardia* may also facilitate colonization by other pathogens. To date, research *in vitro* and *in vivo* has demonstrated differences in the pathogenesis of *Giardia* isolates, but has failed to clearly establish whether these differences are assemblage-specific. [10], [50], [54], [69], [70].

Data from this study also demonstrate that *Giardia* infections reduce the expression of pro-inflammatory mediators not associated with PMN recruitment, in an isolate-dependent manner. *Giardia* NF infections attenuated colonic expression of IL-6 and the related cytokine LIF induced following i.r. TcdAB, whereas *Giardia* GS/M did not. Moreover, co-incubation of *Giardia* NF trophozoites with inflamed human intestinal mucosal biopsy tissues *ex vivo* also resulted in attenuation of supernatant and tissue IL-6. The ability of *Giardia sp.* to modulate IL-6 during *in vivo* infection remains incompletely understood. These observations are consistent with previous studies that have shown that animals infected with *Giardia muris* display decreased IL-6 levels in jejunal tissues 5 days post-infection [71], while mice infected with *Giardia* GS/M display elevated IL-6 intestinal tissue levels [72]. The release of IL-6 and the related cytokine LIF can induce the expression of acute phase response proteins, including CRP [73], [74]. As Tanzanian children infected with *Giardia* were found to have lower serum CRP than their non-infected counterparts [22], future studies should examine whether *Giardia* infections can modulate the acute phase response in a strain-dependent manner, and establish whether the parasite-mediated attenuation of IL-6 expression is involved in this process.

The present findings illustrate that *Giardia* NF infections attenuate colonic expression of CCL2 and IL-12p70 *in vivo,* as well as in inflamed human intestinal mucosal biopsy tissues *ex vivo*. CCL2 is an important chemokine for monocytes and macrophages (reviewed in [75]). Conversely, co-incubation of *Giardia* trophozoites with *in vitro* Caco-2 monolayers results in increased mRNA expression of CCL2 [76]. Additional research is required in order to understand how *Giardia* infections may modulate monocyte/macrophage recruitment. IL-12p70 is composed of IL-12p35 and IL-12p40, and is the bioactive form responsible for inducing helper T1 (T_H_1) adaptive immune responses ([77]). Recent research has demonstrated that *Giardia* trophozoites products are capable of attenuating IL-12p70 expression from dendritic cells (DCs) stimulated with bacterial lipopolysaccharide [78]. In contrast other studies found that *Giardia* products may increase DC IL-12p70 expression [79], [80]. More research is required in order to determine how *Giardia* infections may modulate CCL2 and IL-12p70 expression in the context of intestinal inflammatory responses. We have recently shown that *Giardia* cathepsin B (catB) cysteine proteases degrade intestinal epithelial IL-8/CXCL8 and can attenuate PMN chemotaxis [41]. It remains to be determined whether *Giardia* catB proteases are involved in attenuating PMN accumulation *in vivo* and during human giardiasis. Genetic manipulation of the *Giardia* genome with Cre/loxP system has recently been demonstrated [81]. This would be extremely beneficial in determining the anti-inflammatory potential of *Giardia* catB proteases *in vivo.* Whether *Giardia* may exert immune-modulatory effects using other secretory-excretory products apart from *Giardia* catB proteases remains largely unknown. *Giardia* arginine deiminase has been shown to attenuate nitric oxide production from intestinal epithelial cells [82]–[84], to inhibit intestinal epithelial proliferation [85], to induce expression of IL-12p70 from DCs [80], and to inhibit T-cell proliferation [84], however these effects have yet to be characterized *in vivo.*

In conclusion, results from this study demonstrate that *Giardia* infections can attenuate granulocyte infiltration and expression of PMN chemokines in an isolate-dependent manner in an *in vivo* model of infectious colitis. *Giardia* trophozoites were also able to reduce the expression of a variety of inflammatory mediators released from inflamed colonic mucosal biopsy tissues from CD patients. These findings establish the strain-dependent anti-inflammatory properties of *Giardia* in the intestine.

## Supporting Information

Figure S1 *Giardia* NF infections *in vivo* do not attenuate colonic expression levels of IL-1β, IL-5, CXCL10, or CCL11 during *C. difficile* TcdA/TcdB-induced colitis. Male C57BL/6 mice, aged 4 to 6 weeks, were infected with *Giardia* NF trophozoites. 7 days post-infection, animals were given 100 µg of TcdA/TcdB or PBS i.r. and, after 3 hours, animals were euthanized. Levels of (A) IL-1β, (B) IL-5, (C) CXCL10, and (D) CCL11 were compared between *Giardia* NF-infected animals and uninfected animals given i.r. 100 µg of TcdA/TcdB or PBS. Values were determined via bead-based cytokine assay. All data are representative of two independent experiments (n = 5–11/group) and represented as mean ± SEM. n.s. = not significant * p<0.05.
(TIF)

Figure S2 *Giardia* NF infections *in vivo* do not attenuate colonic expression levels of inflammatory mediators not increased during *C. difficile* TcdA/TcdB-induced colitis. Male C57BL/6 mice, aged 4 to 6 weeks, were infected with *Giardia* NF trophozoites. 7 days post-infection, animals were given 100 µg of TcdA/TcdB or PBS i.r. and, after 3 hours, animals were euthanized. Levels of IL-1α (A), IL-2 (B), IL-7 (C), (D) IL-9, (E) IL-10, (F) IL-12p40, (G) IL-15, (H) IFNγ, (I) CCL3, (J) CCL4, (K) CXCL9 and (L) VEGF were compared between *Giardia* NF-infected animals and uninfected animals given i.r. 100 µg of TcdA/TcdB or PBS. Values were determined via bead-based cytokine assay. All data are representative of two independent experiments (n = 5–11/group) and represented as mean ± SEM.
(TIF)

Figure S3 *Giardia* GS/M infections *in vivo* upregulate colonic expression levels of several inflammatory mediators not increased during *C. difficile* TcdA/TcdB-induced colitis. Male C57BL/6 mice, aged 4 to 6 weeks, were infected with *Giardia* GS/M trophozoites. 7 days post-infection, animals were given 100 µg of TcdA/TcdB or PBS i.r. and, after 3 hours, animals were euthanized. Levels of (A) IL-2, (B) IL-5, (C) IL-15, (D) IL-17, (E) IFNγ, (F) CXCL9, (G) CCL4, and (H) VEGF were compared between *Giardia* GS/M-infected animals and uninfected animals given i.r. 100 µg of TcdA/TcdB or PBS. Values were determined via bead-based cytokine assay. All data are representative of two independent experiments (n = 5–11/group) and represented as mean ± SEM. * p<0.05.
(TIF)

Figure S4 *Giardia* GS/M infections *in vivo* do not modulate colonic expression levels of CCL3 and CCL11 during *C. difficile* TcdA/TcdB-induced colitis. Male C57BL/6 mice, aged 4 to 6 weeks, were infected with *Giardia* GS/M trophozoites. 7 days post-infection, animals were given 100 µg of TcdA/TcdB or PBS i.r. and, after 3 hours, animals were euthanized. Levels of (A) CCL3 and (B) CCL11 were compared between *Giardia* GS/M-infected animals and uninfected animals given i.r. 100 µg of TcdA/TcdB or PBS. Values were determined via bead-based cytokine assay. All data are representative of two independent experiments (n = 5–11/group) and represented as mean ± SEM. * p<0.05.
(TIF)

Figure S5 *Giardia* GS/M infections *in vivo* do not attenuate colonic expression levels of inflammatory mediators not increased during *C. difficile* TcdA/TcdB-induced colitis. Male C57BL/6 mice, aged 4 to 6 weeks, were infected with *Giardia* GS/M trophozoites. 7 days post-infection, animals were given 100 µg of TcdA/TcdB or PBS i.r. and, after 3 hours, animals were euthanized. Levels of (A) IL-1α, (B) IL-7, (C) IL-9, (D) IL-10, (E) IL-12p40, and (F) TNFα were compared between *Giardia* GS/M-infected animals and uninfected animals given i.r. 100 µg of TcdA/TcdB or PBS. Values were determined via bead-based cytokine assay. All data are representative of two independent experiments (n = 5–11/group) and represented as mean ± SEM.
(TIF)

Figure S6 *Giardia* NF trophozoites do attenuate supernatant levels of several inflammatory mediators released from descending colon mucosal biopsy tissues. Human descending colon mucosal biopsy tissues obtained in areas of active inflammation from patients with active Crohn’s disease (CD) were incubated with 5.0×10^6^ *Giardia* NF trophozoites for 6 hours. Supernatant levels of (A) IL-1ra, (B) IL-1β, (C) IL-2, (D) IL-12p40, (E) IL-13, (F) IL-15, (G) IFNγ, and (H) TNFβ were determined via a bead-based cytokine assay. All data are representative of three independent experiments (n = 4–6/group) and represented as median with the range. n.s. = not significant.
(TIF)

Figure S7 *Giardia* NF trophozoites do attenuate levels of several inflammatory mediators released from descending colon mucosal biopsy homogenates. Human descending colon mucosal biopsy tissues obtained in areas of active inflammation from patients with active Crohn’s disease (CD) were incubated with 5.0×10^6^ *Giardia* NF trophozoites for 6 hours. Tissue homogenate levels of (A) IL-1ra, (B) IL-1β, (C) IL-2, (D) IL-12p40, (E) IL-13, (F) IL-15, (G) IFNγ, and (H) TNFβ were determined via a bead-based cytokine assay. All data are representative of three independent experiments (n = 4–6/group) and represented as median with the range. n.s. = not significant.
(TIF)

Figure S8 *Giardia* NF trophozoites do attenuate supernatant levels of several growth factors released from descending colon mucosal biopsy tissues. Human descending colon mucosal biopsy tissues obtained in areas of active inflammation from patients with active Crohn’s disease (CD) were incubated with 5.0×10^6^ *Giardia* NF trophozoites for 6 hours. Supernatant levels of (A) FGF, (B) Flt-3L, (C) PDGF-AA, (D) PDGF-BB, and (E) TGFβ were determined via a bead-based cytokine assay. All data are representative of three independent experiments (n = 4–6/group) and represented as median with the range. n.s. = not significant.
(TIF)

Figure S9 *Giarida* NF trophozoites do attenuate levels of several growth factors released from descending colon mucosal biopsy tissue homogenates. Human descending colon mucosal biopsy tissues obtained in areas of active inflammation from patients with active Crohn’s disease (CD) were incubated with 5.0×10^6^ *G. duodenalis* NF trophozoites for 6 hours. Tissue homogenate levels of (A) FGF, (B) Flt-3L, (C) PDGF-AA, (D) PDGF-BB, and (E) TGFβ were determined via a bead-based cytokine assay. All data are representative of three independent experiments (n = 4–6/group) and represented as median with the range. n.s. = not significant.
(TIF)
